# The Prison Economy of Needles and Syringes: What Opportunities Exist for Blood Borne Virus Risk Reduction When Prices Are so High?

**DOI:** 10.1371/journal.pone.0162399

**Published:** 2016-09-09

**Authors:** Carla Treloar, Luke McCredie, Andrew R. Lloyd

**Affiliations:** 1 Centre for Social Research in Health, UNSW Australia, Sydney, Australia; 2 Inflammation and Infection Research Centre, School of Medical Sciences, UNSW Australia, Sydney, Australia; Curtin University, AUSTRALIA

## Abstract

**Aim:**

A formal Needle and Syringe Program (NSP) is not provided in Australian prisons. Injecting equipment circulates in prisons as part of an informal and illegal economy. This paper examined how this economy generates blood-borne virus (BBV) risk and risk mitigation opportunities for inmates.

**Method:**

The HITS-p cohort recruited New South Wales inmates who had reported ever injecting drugs and who had a negative HCV serological test within 12 months prior to enrolment. For this study, qualitative interviews were conducted with 30 participants enrolled in HITS-p. Participants included 10 women and were incarcerated in 12 prisons.

**Results:**

A needle/syringe was nominated as being typically priced in the ‘inside’ prison economy at $100-$150, with a range of $50-$350. Purchase or hire of equipment was paid for in cash (including transactions that occurred outside prison) and in exchange for drugs and other commodities. A range of other resources was required to enable successful needle/syringe economies, especially relationships with visitors and other prisoners, and violence to ensure payment of debts. Strategies to mitigate BBV risk included retaining one needle/syringe for personal use while hiring out others, keeping drug use (and ownership of equipment) “quiet”, stealing used equipment from the prison health clinic, and manufacture of syringes from other items available in the prison.

**Conclusions:**

The provision of prison NSP would disrupt the inside economies built around contraband needles/syringes, as well as minimise BBV risk. However, any model of prison NSP should be interrogated for any unanticipated markets that could be generated as a result of its regulatory practices.

## Introduction

The risk of acquiring blood borne viruses (BBVs) such as hepatitis C (HCV) and HIV via injecting drug use in prison is well established [1,2], given the lack of access to sterile equipment for drug injecting in most prisons in the world [3,4]. Indeed, prison needle exchange remains a highly controversial program even after a 20 year history [5]. Drug injection continues to occur in prisons, albeit at a lower rate than in the community [6], particularly when opiate substitution treatment is available [7], but with an increased likelihood of sharing injecting equipment [8]. This means that prisoners have developed ways to acquire or manufacture equipment and to access illicit drugs for injection. As with any contraband in prisons, the limited supply of needles/syringes for drug injection opens up the possibility of an informal economic system around the distribution of this equipment. What has not been examined previously is how this economy impacts on prisoners’ abilities to minimise BBV transmission risk.

In prisons without a formal needle exchange program to deliver sterile equipment, the opportunities for inmates who inject drugs to minimise BBV risk are limited to strategies such as not injecting, using only sterile equipment, or attempting to clean the equipment between uses. While the cleaning of used equipment has been described as sub-standard in community settings [9], it is particularly difficult to achieve in prison where cleaning products may not be available or may be difficult to access and prison inmates may fear detection by corrections officers [10,11]. Other strategies to prevent BBVs available to inmates in NSW prisons include access to condoms via vending machines and all inmates at risk of BBVs are offered hepatitis B vaccination [12].

There is little research which has examined the various competing risks that must be negotiated by individuals to minimise BBV risk in prison. A risk environment framework emphasises the mechanisms by which social, economic and political institutions shape health inequalities, including those related to service access and decisions about BBV risk and injecting practice [13]. In community-based research, the literature has examined numerous factors within this risk environment framework across diverse socio-political settings [14]. However, the literature concerning risk environments within prison is much smaller. Some work has highlighted the limitation of epidemiological data in understanding the social relationships that facilitate risk of transmission in relation to prison tattoos [15]. Further and in relation to violence in prison, other authors have called for a greater emphasis on situational factors, rather than reproducing understandings based on individual level factors [16]. Economic influences on injecting practice and BBV risk has received little attention in the prison environment.

Obtaining sterile needles/syringes requires that inmates participate in the informal prison economy. An informal economy in prison provides new opportunities, such as a means to earn additional income or the use of contraband as currency [17]. Other writers have also suggested that informal economies generate rules and regulations that govern inmates behaviours and relationships [18,19]. The ways in which inmates participate in these informal economies can also bring specific risks, especially of violence or victimisation, if they are unable to repay debts [20].

While there has been considerable attention paid to drug consumption in prisons, there has been little focus on the economies, supply chains and distribution of drugs [21], and less that has focused on injecting equipment economies, supply and distribution [22]. Ethnographic research inside prison or qualitative research using interviews with former prisoners, have identified some common features of prison drug economies. Resources based in social networks are required to maintain such economies including the means to access drugs via visits from outside or packages thrown over prison walls (both requiring contacts on the outside with their own resources to acquire and deliver drugs) or importation by the inmate on entry to prison [22]. The capacity to inflict violence or arrange others to inflict violence (sometimes via payment in drugs) is required to ensure drug debts are paid and no other dealer takes on one’s market [21,23]. While also noting the importance of informal rules in a prison drug economy, a study in Norway highlighted a culture of sharing, rather than selling, drugs [24]. The one study examining prison markets for injecting equipment noted that, like drugs, equipment has capital that attracts trade in goods and services and reciprocal exchanges [22]. The author notes that, unlike drugs, injecting equipment is more difficult to smuggle into prison, and that its reusable nature and scarce availability means that it is less likely to be disposed of voluntarily. Hence, it is important to understand how to promote safer injecting in prison “within this trading context” (p61).

The aim of this research is to contribute to understanding how safer injecting, or BBV risk mitigation, is influenced by the prison market for injecting equipment. While the literature regarding drug markets in prison can provide some insight, the nature of the two commodities is different (drugs being entirely consumable) and their role in BBV transmission is not comparable (drugs per se have no role in BBV transmission). There is only limited literature regarding how sterile equipment is acquired by inmates and the means by which it circulates through prison. There has not been detailed analysis of the influence of the informal economy for injecting equipment on BBV risk and risk mitigation. In this paper, we examined how prisoners negotiate BBV risk in an environment in which the key tool for prevention is part of an informal and illegal economy.

## Methods

This qualitative study was conducted as part of a larger prospective cohort study of male and female inmates examining HCV transmission rates and associated risk factors. Participants enrolled in the Hepatitis C Incidence and Transmission Study in prisons (HITS-p) cohort were eligible for this qualitative study. The HITS-p study is a prospective cohort of HCV-uninfected inmates who report injecting drug use. The cohort was established in 2005 and was conducted in 30 prisons across the state of New South Wales, Australia [2,25]. Appropriate human research ethics committees (Corrective Services NSW, Justice Health and Forensic Mental Health, and the University of New South Wales) provided approval for the HITS-p cohort and for this project. Eligibility criteria for the HITS-p cohort included: being aged 18 years or above, reporting a history of injecting drug use at any time in the past and having a documented negative anti-HCV test result in the 12 months prior to enrolment. Exclusion criteria included: anti-HIV-antibody positive status, pregnancy, insufficient English or current psychiatric disorder to preclude consent. Recruitment occurred via posters at prison health clinics, word of mouth among inmates and via research nurses. Participants were monitored every three to six months for HCV antibodies and viraemia, and via interviewer-administered questionnaire to record behavioural risk practices (particularly, injecting drug use, tattoos and fights). While the cohort data can examine relationships between self-reported behaviour, serostatus and other factors, these data cannot account for the complex and inter-related nature of practices and environments surrounding HCV risk (and prevention strategies) among prison inmates. An interview-based method was chosen to allow participants to fully discuss and explore the practices and settings of injecting drug use in prison.

During 2013–2014 participants in HITS-p were invited to participate in this qualitative study with in-depth interviews conducted by a research nurse (LM) well known to them as a result of their participation in the cohort study. Inmates were informed of the qualitative study by the research nurse and offered the opportunity to participate. The nurse explained the purpose of the study and the inmates’ right to accept or decline the offer. When interviews were scheduled and the inmate attended, the nurse reiterated the ethical principles of informed consent and confidentiality, withdrawal without penalty and the importance of avoiding discussion of specific incidents requiring legislated mandatory reporting to authorities (see S1 File. Interview Schedule). Written informed consent was obtained. Qualitative interviews lasted 30–70 minutes. Participants were interviewed once. At the conclusion of each interview, participants were offered written information about HCV, an opportunity to discuss any further issues with the research nurse, and information about access to the Prison Hep C Infoline. Participants received AUD$10 for their participation in the interview through the approved prison inmate banking system to compensate for their time and effort in completing the research interview.

Recruitment was conducted in as many of the participating HITSp prisons as was feasible. The frequency of prisoner movements between prisons in NSW and between prison and the community is high. Previous work from the HITSp study showed that cohort participants had moved locations (to another prison or to the community) a median of 17 times [26] during 2005–2012. This means that participants would likely be able to comment on their experiences across a range of environments allowing analysis of those experiences that were tied to particular settings or types of settings (such as related to security classification or other features of the prison environment) and those similar across settings.

The interview schedule included topics such as: risk (what risks are perceived by prison inmates; what risks can be compromised or negotiated and what cannot); HCV awareness; HCV information sources; susceptibility to HCV; and, injecting drug use, tattooing and violence (including details of how, where, when, with whom these activities occur; how equipment is sourced; decisions/influences on safety and practice). The interview was guided to avoid disclosure of specific details of *individual* injecting or risk behaviour events (including dates, names of individuals involved and specific locations as such events may constitute criminal behaviour and warrant reporting to authorities). Demographic information was collected from all participants.

Interviews were audio-taped and transcribed verbatim. Transcripts were checked for accuracy against recordings and de-identified. The data was then read closely and a number of themes identified as relevant to the research questions. The research team then collaborated on the construction of a ‘coding frame’–a set of organising, interpretive themes to aid analysis. The coding frame was then used to organise interview data within NVivo 9. Each aspect of the thematic analysis, that is the interpretations and meanings drawn from the interview data, was critically examined and summarised (along with supporting quotes). Analysis was informed by both a deductive and inductive approach [27]. That is, participants’ narratives were examined for issues relating to the economies surrounding needles/syringes in prison. This involved analysis of the price attached to this equipment and other material effects that could be bartered or exchanged for access to, or ownership of, equipment. We also considered the economies of equipment in broader terms and examined other resources that are implicated in these exchanges, such as issues relating to relationships that facilitate the economy and the personal resources (skills and willingness) to undertake these exchanges. The influence of the prison environment on various needle/syringe economies was also examined, particularly the influence of the security classification of the prison. Finally, we examined the ways in which the resultant economies provided opportunities for mitigation of BBV risk in relation to drug injection. It is important to note that there are multiple factors shaping the ways in which needles/syringes circulate in prison and the BBV risks that these produce. Hence, we have use the plural “economies” to indicate the multiple interactions of markets, vendors, investors and currency. All currency referred to is in Australian dollars. The amounts quoted can be viewed in reference to an amount of $50 which is a typical price reported in the community for a “cap” of heroin (used for a single injection) [28]. Quotes are presented by participant number, gender, age and frequency of injecting reported at last behavioural survey.

## Results

Thirty inmates participated, including 10 women. Participants ranged in age from 22 to 67 years, with most (n = 24) aged 35 years or less. Seven participants identified as Aboriginal or Torres Strait Islander. Of this group, all of whom reported a history of injecting drug use, 14 had not been exposed to HCV at the time of interview, 8 had chronic HCV infection and 8 had incident infection. None had HIV infection. The majority (n = 19) reported 10 years or less of formal education. At the time of interview, 10 participants reported no recent injecting, six reported injecting at a frequency of less than monthly, three more frequently than monthly, three more than weekly, three daily and five more than daily as indicated by their responses to behavioural surveillance surveys. Participants were recruited from a total of 12 prisons (including all three female prisons in NSW).

### How needles/syringes are acquired

Four main means of acquiring needles/syringes were mentioned by participants: through visits; “drops” of equipment in or around the prison campus; bringing equipment to prison from the community or when transferred between prisons; and, stealing equipment from the health centre. A fifth strategy, obtaining equipment via staff, was alluded to in indirect terms (as it was considered a very sensitive topic) and will not be explored in detail here.

Participants described a range of ways in which equipment could be obtained during prison visits. This equipment may not have been previously used for injection but may no longer be sterile because of the manipulation or modification required for its smuggling into prison. Visitors were asked to bring in equipment that had been “cut down” and prepared for the inmate to swallow or secrete in underclothing: that is, the barrel of the syringe shortened, the cap of the needle taped on and the equipment wrapped in the finger of a disposable glove, condom or balloon. Visitors brought equipment in to prison on their body or disguised within hairclips to avoid detection by metal detectors and other surveillance devices. Participants described swallowing the equipment for later regurgitation or passing if they could not secrete the equipment on their person. Others noted that inmates employed to clean the room after visits could retrieve equipment taped to the underside of tables. Obtaining equipment through visits was described as more difficult in maximum security prisons in which inmates were required to wear overalls (making access to underclothing difficult and conspicuous), and were required to submit to invasive searches after visits.

Drops were arranged via external contacts to enable a package to be left at a specific site or thrown over prison walls (for example, disguised within tennis balls). At lower classification prisons, inmates on release or working in the community retrieved packages from pre-designated areas.

Movements between community and prison, and between prisons, were opportunities for the importation of equipment. If individuals knew they were to be incarcerated, they could prepare by secreting equipment. With equipment typically easier to access in lower security prisons, inmates who knew they were being moved from a lower to a higher security prison would attempt to bring equipment with them. These strategies also had the potential to go awry, with unanticipated transfers between prisons leaving participants without the opportunity to retrieve their equipment. In this case, the inmate would arrive at the new prison at the bottom of the pecking order of the needle/syringe economy.

They get in trouble at C-classo gaols [minimum security], lose their classification, come back to maxo [maximum security] and they’ll smuggle some shit back with ‘em, you know. They’ll bring back syringes … you see stuff make its way in, into the hands of, you know, of blokes in maxo by that means too. (6, male 29 years, inject less than monthly)

I left another gaol where I left my fits… I didn’t have access to where they were buried. … Before I was shipped out, I couldn’t get access to ‘em and I was moved on a truck out here, and I couldn’t get to ‘em. … So now I’m here with no fit. (8, female, 33 years, inject less than monthly)

Inmates described stealing sterile and used equipment from the health clinic. This could be done by inmates during their own consultation (including when receiving HCV treatment) or by an inmate working in the clinic area (a service also requiring a fee). Although inmates were accessing equipment from needle disposal bins, this risk was rationalised by the assumption that these needles/syringes had been used for intra-muscular injections, carrying a lower risk than equipment used for intravenous injections: “that’s how you try and not catch hep C” (8, female, 33 years, inject less than monthly). Mass vaccination periods were identified as particularly fruitful times for this mode of equipment access.

Just being in the right place at the right time or the nurse not being in the right place at the right time—yeah they just swipe it. Yeah they rip [the sharps disposal container] open and try and get one out of that or something (31, male 39 years, inject more than weekly)

I met a young bloke and I gave him a couple of smokes of pot, you know. Just a couple of cones and that. And became friends with him, and then I said, “Hey, listen.” This was around the time when the swine flu epidemic came out and I said, “Hey, listen,” you know, “there’s, they’ve got thousands of, of syringes up there.” 'Cause they had to vaccinate the [whole] gaol. And they did, they had two boxes, call ‘em 1,000. I said, “Look … get into the fridge and I’ll pay ya.” … And so he did. He was bringin’ nearly two back a day for me. … I was sellin’ them for like 60 bucks [dollars] each, you know. But, at one stage, it was somethin’ like 20, 25 syringes just in our wing alone. (6, male, 29 years, inject less than monthly)

### The cost of needles/syringes

The price of needle/syringes quoted by participants was, unsurprisingly, dependent on supply and demand factors. The security classification of the prison and the location of the prison (drugs and equipment being in more plentiful supply in metropolitan compared with regional sites) were commonly cited as impacting on price. The lowest cost for a sterile needle/syringe was $50 and the highest was $350. More commonly, participants spoke of amounts around $100-$150. While sterile needles commanded the higher price, the cost of used needle/syringes was described as $40. Participants noted these extreme prices for a commodity that can be obtained for free in the community.

I’ve been to C-classo gaols before and the syringes that are available … you can buy them in a packet and they’re very cheap. And they’re not modified. Like they’re not cut down or anything like 'cause of how easy they are to smuggle in, you know. So it’s a lot safer. … I’ve been in C-classo gaols before where I’ve used like I’ve used on the outside. I’ve used a syringe and, you know, then thrown it out because I can go get another new one. (6, male, 29 years, inject less than monthly)

I mean I could have sold that for 200 bucks. It’s absolutely ridiculous how much you can sell fits in here for. And yet that’s the thing: if it’s brand new and clean, yeah, you could sell it for 200 bucks. (1, male, 29 years, inject more than once per day)

The high prices paid by inmates show the inherent value of sterile equipment. Why inmates would want sterile equipment is embedded in notions of safety. The reasons for selling equipment, are unsurprisingly, embedded in economic drivers that can pertain both to the inmate’s economic situation and the needs of their family in the community, particularly when other ways to make money were unavailable.

[My girlfriend] didn’t have a choice….

It was money that we didn't have that I could make. I could sell a fit, you know, a brand new fit for $200, $300 and go to the hospital, and get 10 for nothing. … That was good money and I’d tell her, “Do it!” And she would have done it, you know. She was bringing in pills every weekend. I was making a killing. More money than I make on the outside.

Interviewer: How do you, how do you know it’d be 200 bucks?

I put a price on it. If they want it, they’ll buy it. They’ll take it (11, male, 51 years, inject less than monthly)

Without sterile equipment entering the market, used equipment remains in circulation for longer periods. Some participants noted that they were aware of equipment that had been in circulation for two or three years (2, male, 23 years, inject more than once per day). Even when equipment was dangerously worn, inmates would “hold onto it for fuckin’ Christ knows how long” (10, male 51 years, inject less than monthly) for fear of not having access to equipment at all.

There’s not even numbers on it, on the fit. So where the new ones have got the numbers and they’ve just got nothing, and these needles are bent, you know, like side-wards. They’re, they’re blunt as, you know, it’s like sticking a frigging nail in your arm. … And that’s what gets around. And that’s where I worry about the, the little tip breaking off into the vein. And I, I tell that to girls, you know. You should just throw ‘em away when they get like that. But, because they’re, they’re users, basically, they won’t throw ‘em away. (24, female, 24 years, no injecting reported)

### Paying for needles/syringes

There is no access to cash as a currency in the prison system to pay for needles/syringes. Participants described a number of different ways that goods and services circulated, within and external to the prison, to pay the high price of a sterile needle/syringe. Exchanges of cash in the community, prisoners paying for the vendor’s “buy-ups” (purchases from the prison store), trading of goods (such as sunglasses and thongs [rubber sandals]), and less frequently sex, were described as aspects of the needle/syringe economies.

Between bank accounts. … obviously $350 is a lot of money so that cannot be organised. That, can’t be accessible inside gaol. So I then arrange for you to put the 350 into an outside account which I have access to or my people outside have access to. That’s how that transaction’s done. (9, male, 35 years, inject more than weekly)

While the above relates to the outright purchase of a sterile needle/syringe, other arrangements were apparent—best described as “renting” of equipment. In some cases, inmates arranged importation of a number of needle/syringes so that they could keep one for personal use and rent out the remaining pieces for use by others. In this way, the owner of the equipment minimises the risk of BBV to themselves, while providing a sought after commodity for others and profiting from this service.

They bring a few cut-downs in and the, the owner will keep one and sell two, you know, to make more profit, more money. … They won’t let anyone use their own fit. Smart thinking. (24, female, 24 years, no injecting reported)

The transactions involved in renting equipment were frequently based on the offer of drugs, including tobacco, as payment for use of the equipment. Besides tobacco and cannabis for smoking, owners of needles/syringes for rent could also command shares of drugs for injection. In some cases, this service included the owner sharpening and cleaning the equipment between renters.

if you’ve got the needle, then the girl that gets the drugs will come up and they’ll say, “Can I use your needle?” And then that person will say, “Well what are you gonna give me for the needle?” They’ll say, “I’ll go you halves.” So that’s, so having a syringe is like having this, this thing that just attracts all the injectable drugs, 'cause they wanna use that needle. But that person’s not gonna give it to you for free, you've gotta shout [share] as well. (12, female, 26 years, no injecting reported)

lot of guys rent 'em as well, … I knew of a guy who used to actually call his needle the 'Rent-a-Hep'. … Yeah, he used to just charge half a packet of White Ox [tobacco] to use the needle. … And then you've gotta bring the needle back. That was in maximum security 'cause they're a bit harder to get. Yeah. Used to maintain it. He'd sharpen it up for 'em and everything, you know. Clean it. And then send out the Rent-a-Hep as he used to call it. … He was doin' it for a long time. He was makin' a fuckin' probably 100 bucks a week from it. 'Cause all the junkies want to borrow it. He's a big guy though. To, to be able to do something like that you've gotta have muscle, you know. You can't be just a, a weakling or an underling to do something like that. …They've gotta know that they're gonna have their head jumped on unless it's been returned (18, male 27 years, inject more than once per day)

## Resources involved in needle/syringe economies

At face value, the needle/syringe economy is based on the availability of equipment (sterile and used) and the resources to pay for it (notably cash in the community, goods, tobacco and other drugs). However, in a closed environment with a strong culture of control of contraband and illegal associations, other resources are required to maintain the circulation of needles/syringes.

Relationships with people willing to undertake the risk of bringing needles/syringes into prison is a key resource required for this economy to function. These relationships must be seen in light of what could be for many inmates, a long history of criminal behaviour and possible estrangement from family and friends in the community. Bringing equipment into prison brings risks for the visitor as well as the inmate who may have privileges withdrawn or acquire additional charges added to their record, thus extending their sentence.

not everyone has the people on the outside to do it because they burnt everybody they fucking know, but the ones that do have people out there, … they eventually get caught anyway. They never have a full run their whole lag [sentence], their visitors will get caught, they will get barred, it’s only a matter of time but until that happens by then if he gets caught someone else has rocked up (29, male, 33 years, no injecting reported)

Relationships with other inmates were key to establishing and maintaining these economies. Participants described a range of relationships that were useful in gaining access to equipment including inmates with visitors prepared to smuggle equipment into prison and inmates who were diabetic or required other injectable treatments, including HCV treatment. Relationships with prisoners with work roles (“sweepers”) were also relied upon to access equipment hidden in visiting areas or to steal equipment from health clinics. The character of these relationships was also described as important. Participants described the process of renting a needle/syringe to another inmate as a “pick and choose” process dependent on reputation with “nobodys” generally excluded from renting equipment.

you have a reputation sort of thing, you know, whether it’s bad or whether it’s selling drugs. You have a reputation in here. So, if you’ve got the good reputation and you have connections, then that makes it a lot easier for you to get to where you want. … And to access what you want sort of thing. (1, male, 29 years, inject more than once per day)

They might want to borrow it but the bloke who owns it might not lend it to them so they don’t end up using it and that happens a lot, they pick and choose who they fucking lend their needle to (29, male, 33 years, no injecting reported)

For participants who needed to rent equipment, targeting of specific people was a strategy employed with varying levels of obvious intent. Some participants noted that they observed such predatory behaviour in others and that this was an obvious strategy used when an upcoming “drop” became common knowledge. Others sought to cultivate friendships with strategically significant inmates over a longer time.

Interviewer: So how did you get your hands on that diabetic to get the needle?

Well my first step was finding myself a diabetic. And then yeah, becoming, becoming mates with them and–

Interviewer: So you became mates with them on purpose?

Not, not just for that fact but, you know, I just, just, yeah, just … brought, brought them into my circle, so to speak, but on the proviso of making a, a friendship too; not, not just for my ill-gotten gains. But, you know … (3, male, 31 years, inject more than once per day)

Like there’s a lot of girls in here that would pretend to be somebody’s friend for that week because they know they’re getting a drop through visits. So there’s a lot of girls in here that do that 'cause they know this certain person’s getting drugs that weekend, so they befriend her. (8, female, 33 years, inject less than monthly)

In prison, consorting with officers is seen as a violation of the prison code, which casts relationships between inmates and authority with disdain. However, in efforts to access equipment and drugs, relationships with corrections officers were a strategic necessity for some participants to enable access to other areas of the prison where such commodities may be stored.

like I try to treat officers with at least a bit of respect unless they just, they've done their dash with me, because I need to do things as well like especially if you're, if you're in the drug scene and that. Normally you've gotta get out of the wing to go and get fits and get drugs, and that, so normally you've gotta keep a good relationship with them. … So that's my, my end of the bargain is to keep good with them. (19, male, 27 years, inject more than once per day)

This participant went on to explain that he has a limited amount of time after his working day to access drugs and equipment. At the time of interview, there was no equipment located in his own prison wing. Hence, he needed to maintain relationships with prison officers to move between areas in the prison before lock down. This participant also noted the need to maintain relationships with other inmates to manage these transactions with limited time and resources.

'Cause I work, I inject after work. … If I have to borrow a fit then I have to do it early enough to get it back to them. … our wing doesn't have a fit right now. So one of us has to go out and get one. Sometimes it's, like say my mate gets the drugs, I'll go and get the fit. Say I've got the drugs, he'll go and get the fit. So one of us is always [the runner] throwing a little somethin' in. So, if I bring the drugs, he brings the fit. You go in together. (19, male, 27 years, inject more than once per day)

Violence can be a key resource to establish or maintain these relationships. Violence, or the threat of violence, was a key tactic to ensure the timely return of rented needles/syringes of payment of debt. This violence is also driven by the inherent value of the equipment.

if someone finds out they've got a decent fit, they could get stood over for it, bashed for it, you know. … Well a brand new fit in gaol goes from anywhere from 100 to $150 for just like a one mil ultrafine. … That's the value of them in gaol.(18, male, 27 years, no injecting reported)

Besides the social resources required to participate in the needle/syringe economy, individuals’ skills and characteristics were also implicated in these activities. Participants raised a number of risks associated with the selling and exchange of needles/syringes including those imposed by the corrections system (withdrawal of privilege and additional charges; additional scrutiny related to presumed drug use) and those imposed by other inmates (violence and “stand over”). For those who chose to engage in needle/syringe economies, this suggests that they possess the confidence required to undertake subterfuge in a highly scrutinised environment, such as to pass equipment in visits or steal equipment from the clinic, and the skills to negotiate complex and unpredictable relationships with other inmates including the self-control to keep drug use “dark” to avoid drawing the attention of other inmates.

A further skill noted by participants was the ability to manufacture equipment from items available in the prison or via cannibalising broken needles/syringes. Various participants described the ways in which needles/syringes could be made in prison describing “gaol as the mother of invention” (3, male, 31 years, injecting more than once per day) and inmates as “experts at fixing ‘em and doctoring ‘em and fixing them up” (16, male, 27 years, no injecting reported). Common items in prison were co-opted for use in needle/syringe manufacture including the plastic tube of spray bottles (as barrel), rubber thongs (as plunger), cotton buds (as plunger), eye dropper (as barrel) plastic packaging from paint brushes (as barrel) with blu-tac, muffler putty or melted plastic used to attach these items to a needle tip (which can be sharpened after use).

## Discussion

Organised and profitable informal prison economies for needles/syringes are made possible because this equipment is not provided in these settings in contrast to the way in which it is provided, typically for free, in the community. Little attention has previously been paid to the influence of the economies built around the importation, sale and use of needles/syringes in prison on BBV risk and risk mitigation. Other authors have argued for more attention to the economic aspects of inmates’ experiences to understand and respond to violence among inmates [20] or to understand the drug market to reduce harms [23]. The data presented here argue for similar attention to be paid to the economic issues that drive the risks and risk mitigation opportunities associated with BBV transmission and injecting in prison. This is the first paper to report actual costs for needles/syringes and explore more generally the relationship between equipment economies and BBV risk. Furthermore, our findings also highlight the intrinsic importance many inmates place on reducing BBV risk–perhaps surprising in a context where the prevalence of HCV infection is approximately one in three [2] and where the cost of “new” equipment in prison could be between two and seven times the cost of a single dose of heroin in the community.

The data suggest that there are far more BBV risks related to needle/syringe economies than there are opportunities to mitigate against those risks. Nonetheless, a few instances of deliberate or opportune BBV risk mitigation were apparent from participants’ accounts. Participants with sufficient resources to procure or buy a number of sterile needles/syringes and retain one for their exclusive use (while selling or renting others) placed themselves in the safest situation, especially when combined with the relationship-management strategy of keeping knowledge of their equipment “dark”, or unknown to others. A novel finding was the unanticipated BBV risk management strategy revealed in the logic underpinning stealing equipment from the health clinic. Inmates assumed that equipment found in the sharps disposal waste of health clinics had been used for intra muscular injections and not necessarily for a BBV-infected patient, and hence retaining less blood than needles/syringes used intravenously. The “do it yourself” (DIY) industry for the manufacture of needles/syringes has been noted before [29]. DIY equipment may result in lower BBV risk if the barrel and plunger is made of other items. However, no participant revealed any other source of needles for DIY efforts than previously used equipment. Further, the use of items such as thongs and cotton buds will introduce other risks (such as infections or injury) as these items cannot be sterilised or may be contain contaminants from their original purpose.

Previous scholarship in the prison environment has noted the importance of relationships as a resource for organising and managing illegal activities in prison [21]. As previously noted, prison inmates are “already skilled at financing and obtaining illegal substances; managing lieutenants, adversaries and turf; and eluding social control agents” [17] (p. 158). That these skills have been turned to tradecraft of needle/syringe economies is no surprise. Indeed, while participants noted a number of risks and possible penalties associated with this market, the high financial profit of this industry (and the direct benefits in terms of drug injection) and desire of inmates to use “new” equipment drove others to actively participate as vendors, customers or both, depending on circumstance.

The potential for violence warrants particular consideration. The specific contribution of needle/syringe economies to prison violence has not been recognised previously. While violence associated with drug use has been noted [23,30], our data revealed specific instances of violence associated with needle/syringe economies. This violence operates in line with the prisoner code [31] suggesting that even if the violence is sufficient to warrant removal of an inmate to protective custody, no information about the violence is revealed to the prison authorities. The provision of formal NSP in prison may reduce violence between inmates as this element of potential conflict is removed.

Prison NSP were first established in 1993. However, the implementation of such programs remains rare, with services offered in approximately 0. 6% of prisons internationally (60 prisons out of more than 10,000) and across 13 countries (compared with 82 countries that operate community-based NSP) [32]. Prison NSP operate via a range of models (distribution of health worker, distribution by peer outreach worker, distribution by external agency, distribution by machine) and in a range of settings including men and women’s prisons, at all levels of prison security and size and with different physical environments [4]. Evaluation of prison NSP shows a range of positive effects including increased institutional safety, no increase in drug consumption or injecting and reduction in risk behaviour [33]. However, prison NSP models that compromise inmate confidentiality (that is, inmates fear being identified as a drug user because they can be seen to use the NSP service) may lead to a lower uptake of the service or the development of unanticipated ways in which the service could be used [5].

This study involved male and female participants recruited from and with experience of a range of prison environments (such as varied security classification) and a range of recent injecting frequency. Participants’ accounts of costs of and obtaining needles/syringes did indicate an association between the security classification of the prison and supply factors (that it is easier to acquire equipment in lower security prisons, resulting in more equipment circulating and lower prices per unit) which could influence BBV risk. Differences in supply aside, participants’ accounts of the organisation and operations of needle/syringe economies were sufficiently similar to enable an overall picture of how these economies operate in NSW prisons. Informal prison needle/syringe economies may produce different opportunities for BBV risk mitigation in response to local factors, including the availability and cost of equipment as driven by supply and demand. These 30 participants reported different levels of injecting frequency in the most recent behaviour survey (from no injecting to more than once per day). Nonetheless, all participants spoke knowledgeably about this economy and no apparent differences (including less knowledge) were apparent amongst those with less frequent injecting. It is also possible that those who inject infrequently now (or during the current sentence) had differing injecting frequencies in the past. These data also raise questions for future research including the differing patterns of needle/syringe economies in men’s and women’s prisons with female inmates report higher lifetime rates of injecting drug use [34] but men are more likely to inject in prison [35]

Scholarly work in relation to BBV risk has long noted the importance of a range of environments and forces, including economic drivers [36,37]. However, the needle/syringe as an economic object in prisons has received scant attention in countries like Australia where equipment access is typically free in the community. This oversight requires redressing in the prison context where the impact of prison regime and regulation are important determinants of prisoner health [38]. The provision of prison NSP would effectively remove the contraband status of needles/syringes and change the ways in which informal needle/syringe economies operate. However, any model of prison NSP should be interrogated for any unanticipated markets or harms that could be generated as a result of its regulatory practices [39].

## Supporting Information

S1 File Interview Schedule. (DOCX)
