# Supplementary material for: The Prison Economy of Needles and Syringes: What Opportunities Exist for Blood Borne Virus Risk Reduction When Prices Are so High?
Source: PLoS One. 2016 Sep 9;11(9):e0162399. doi: 10.1371/journal.pone.0162399 (PMC5017673; doi:10.1371/journal.pone.0162399)
Supplement: S1 File — (DOCX) [file pone.0162399.s001.docx]

**Interview Schedule**

(note this is a complete list of topics to be covered, this list is to ensure that the concepts behind the issues are clear to the interviewer. Issues for discussion will be raised with the participant in conversational style)

Opening

Thank you for agreeing to be part of this

This conversation is focused on issues associated with hepatitis C.

You don’t have to answer any questions that you feel uncomfortable about.

Your name will not be recorded anywhere. We will change any information that identifies you or anyone else

*Emphasise no names needed – and that information should not be provided by participant that would implicate themselves or others in serious illegal activity*

Risk

What do prison inmates worry about?

What types of risks do you see for prison inmates?

What are the most important things for prison inmates to take care of? (ie things they wouldn’t want to jeopardise)

What are things that can be negotiated? Or let go of if necessary?

When you think about injecting, what do you think prison inmates worry about?

What are the risks?

What wouldn’t they compromise?

What would they compromise?

Hepatitis C awareness

How would you describe hepatitis C

- what is it?

- what does it do to people?

- how does someone get it?

- what are the specific ways in which someone can get hepatitis C? What do they have to do to get it?

- how does someone avoid getting hepatitis C?

How common do you think hepatitis C is among people who inject drugs?

How many people do you know have hepatitis C?

Hepatitis C information sources

How have you found out about hepatitis C?

- in prison?

- in the community?

Who do you trust about hepatitis C information? (people and organisations)

Who don't you trust?

Hepatitis C susceptibility

*for those who don't have hepatitis C*

What do you think are your chances of getting hepatitis C?

Do you have worries or concerns about hepatitis C?

*For those who have hepatitis C*

Before you were diagnosed, what did you think about your chances of getting hepatitis C?

Injecting drug use

Can you tell me about a typical injection in prison?

Explore the type of drugs used, how they were used, frequency, who they use with.

- Where do you use? When? By yourself? With others?
- Do you usually share any equipment (use someone else’s used equipment, or pass on your own used equipment?). probe for all equipment.
- Explore where they get equipment, type of equipment they typically access, explore coverage/quantity/access
  - How many people do you estimate used the needle/syringe before you did?
- Do you clean equipment? How do you usually do that? (where do you get cleaning equipment from, what is it, what do you do with it?)
- What do you consider to be safe injecting? Probe safety in relation to self and others
- What influences your decisions about safety? probe:
  - personal (withdrawal, don't care)
  - interpersonal (trust, hierarchies, fear, size/composition of injecting networks)
  - organisational (opportunities, transactions)
  - structural (type of prison)

Tattooing

- What type of body art have you seen done in prison? (tattooing, piercing (temporary or permanent), cutting, scarification).
- How has this been done? probe
  - what types of people do it?
    - how do they become known as the artist? Why do people trust them to do it?
  - what type of equipment is used?
  - who is there when the art is done?
  - what type of decisions about risk are made by people who get body art? What would be important in their decisions?
  - What influences your decisions about safety? probe:
    - personal (withdrawal, don't care)
    - interpersonal (trust, hierarchies, fear, size/composition of injecting networks)
    - organisational (opportunities, transactions)
    - structural (type of prison)
  - Would people be worried about risk for passing on hepatitis C?
    - What types of things could someone who wanted a tattoo do to minimise their risks?

Fighting/Violence

What type of violence have you seen in prison?

What are the situations in which violence occurs?

What are the things that people worry about violence - ie possible outcomes -

Does hepatitis C figure in this?

Demographics

- Age
- Age at first injection
- Length of current sentence
- Is this a first sentence?
- Location (prison)

Closing

Would you like to find out more about hepatitis C?

- If yes, make referral

Thank you for your time

Remind re confidentiality protections.
